# Relationship between Pharmacological Treatment Strategy and Cognitive Function in Geriatric Patients with Atrial Fibrillation

**DOI:** 10.3390/jcm12247724

**Published:** 2023-12-16

**Authors:** Markus Goetze, Tim Knauf, Henning Ebelt

**Affiliations:** 1 Department for Geriatrics, St. Elisabeth Hospital, Bahnhofstrasse 19, 99976 Lengenfeld unterm Stein, Germany; m.goetze@kh-lengenfeld.de; 2 Department of Medicine II, Catholic Hospital “St. Johann Nepomuk”, Haarbergstr. 72, 99097 Erfurt, Germany

**Keywords:** cardiac arrhythmia, anticoagulation, cognition, old aged patient, comorbidities

## Abstract

Background and question: Atrial fibrillation (AF) is the most common cardiac arrhythmia in the total population. The aim of this study is to determine how geriatric patients with AF are treated in terms of rhythm or rate control and whether a relationship between the type of treatment and Mini Mental Status (MMS) can be identified. Methods: In this monocentric, prospective, observational study, data including chronic medication as well as demographic parameters were collected from all patients in a geriatric department between April 2021 and April 2022. A 12-lead ECG as well as the Mini Mental Status were recorded for all patients as part of the admission routine, and a 24 h ECG was performed in selected patients on the basis of clinical indication. Results: At baseline, 715 out of 1914 patients (37.4%) had a known history of AF. Of these patients, 43 patients (6%) were on rhythm control therapy (RHY) and 672 (94%) were on rate control therapy (RATE). No difference in respect to MMS could be detected between RHY and RATE. However, linear regression analyses showed that age, HASBLED score, creatinine serum level, and an existing antiplatelet medication were associated with a negative influence on MMS, whereas oral anticoagulation (OAC) was associated with improved MMS, respectively (*p* < 0.05 for all). Conclusion: The vast majority of geriatric patients with AF are treated with a rate control strategy. Oral anticoagulation is associated with better results in MMS, whereas patients who are treated with antiplatelet medication show worse results in MMS instead.

## 1. Introduction

Atrial fibrillation (AF) is one of the most common cardiac arrhythmias in the general population with a prevalence of approximately 2.5%, although several studies have shown a higher prevalence in the elderly [1]. The lifetime risk is about 1:3 for both women and men [2]. As the general population is getting older and the probability of developing AF increases significantly with age, the prevalence is expected to double in the next 50 years [3].

AF is associated with increased mortality and hospital admissions independent of left ventricular ejection fraction [4]. Furthermore, extracardiac diseases such as depression or cognitive deficits up to dementia have been observed in connection with AF [5], and an increased risk for the development of both vascular and Alzheimer’s dementia has been associated with AF [6]. In addition, AF is the most common cause of ischaemic stroke in people over 75 years of age [7]. As thromboembolic complications are the main factor in the prognosis of patients with AF, therapeutic anticoagulation is of crucial importance, especially in geriatric patients. In a recent analysis, it was shown that the life expectancy of patients with AF has improved in the recent past, but it still remains significantly reduced [8].

Various tests can be used to assess cognitive performance and quantify existing deficits. The Mini Mental Status (MMS) is frequently used and well-established, as it provides a good evaluation of the most important brain functions with comparatively little effort. It is based on different questions and tasks related to temporal and spatial orientation, attention, memory, speech/language comprehension, writing, reading, drawing, and arithmetics, respectively [9].

Systemic anticoagulation is the decisive measure to effectively reduce embolic complications and the associated mortality in patients with AF [10]. Additionally, regarding the treatment of the arrhythmia per se, a distinction must be made between measures to maintain or restore sinus rhythm (rhythm control, RHY) and a strategy which only aims for an adequate and appropriate ventricular rate (rate control, RATE). However, sufficient control of the heart rate is an important therapeutic goal for all patients with AF, both initially and in the long term, in order to prevent the occurrence of complications such as tachycardiomyopathy and to alleviate their symptoms. Accordingly, an adequate rate control is achieved when the mean heart rate at rest is between 80 and 110/min [11,12].

The likelihood of maintaining sinus rhythm depends on several parameters, with the most important of which is the total duration, and length, or number of episodes of atrial fibrillation. However, until a few years ago, several large, randomised trials in the past failed to show that rhythm control has a prognostic advantage over rate control in patients with AF [13,14]. Only recently, the EAST trial was the first to demonstrate the prognostic benefit of an antiarrhythmic strategy for early diagnosed AF [15]. Despite consistent anticoagulation in both treatment groups, stroke was the main event prevented by rhythm control therapy [16]. In older patients, a similar tendency can be assumed with regard to the risk of stroke, but a difference in overall mortality is not found in the synopsis of the available clinical studies [17]. On the other hand, adverse drug reactions might occur more frequently with antiarrhythmic drugs, so no clear therapy recommendation can yet be made in this regard for the elderly population [18].

The aim of the study presented here is to determine how geriatric patients with AF are treated in terms of rhythm or rate control in daily routine. Additionally, it should be analysed whether there is a relationship between the type of treatment (rate or rhythm control, respectively) and the Mini Mental Status (MMS), and whether other factors such as chronic medication are linked to cognitive performance.

## 2. Patients and Methodology

### 2.1. Study Design

The present study is a monocentric, prospective, observational study in geriatric patients, which was conducted in the department for geriatrics at St. Elisabeth Hospital, Lengenfeld unterm Stein, Germany. All inpatient admissions between 1 April 2021 and 1 April 2022 served as the patient collective. Participation in the study was voluntary; the only exclusion criterion was a lack of consent to study participation. A positive vote of the Ethics Committee of Thuringian Medical Association was obtained (reference: 22862/2021/46). The study protocol and the results on prevalence and incidence of AF have been published [19].

### 2.2. Data Collection

Data collection was carried out using the electronic patient records. The following variables were obtained at hospital admission (baseline): height, weight, date of admission, age, sex, Barthel Index, Mini Mental Status (MMS). Creatinine, ALAT, ASAT, INR, bilirubin were measured by venous blood sampling as part of routine treatment and recorded in the study documentation. Information on history of AF and relevant concomitant diseases such as arterial hypertension, heart failure, diabetes mellitus, previous stroke, liver cirrhosis, bleeding history, coronary heart disease (CHD) and other vascular diseases as well as risk factors in health behaviour, such as chronic alcohol abuse and information on chronic medical treatment were taken from the electronic patient records. Detailed information regarding the duration of AF could not be collected. As part of the admission routine, each patient received a 12-lead ECG. In addition, a 24 h ECG was recorded in selected patients based on clinical indication. The CHA_2_DS_2_-VASc and HASBLED score were calculated to assess the risk of stroke and bleeding, respectively. 

Those patients with a history of AF, who either had a history of AF ablation or were on medication with amiodarone, dronedarone, sotalol, or a class I antiarrhythmic drug, respectively, were assigned to the rhythm control group (RHY); all other patients were considered to be under rate control therapy (RATE).

### 2.3. Analysis

All statistical analyses were performed using SPSS (version 29). The level of significance was set at *p* = 0.05. Metric variables are given as mean values with standard deviation. All metric variables were tested for normal distribution by the Kolmogorov–Smirnov test. Statistical comparisons were performed with student’s *t*-test or ANOVA for metric variables, respectively. Differences between categorical variables were analysed by Pearson’s Chi-square. Linear regression analysis was performed to analyse the influence of different variables on MMS. To identify the differences in patient characteristics between the two groups RATE and RHY, propensity score matching was used were all base line variables that were found to be different between the two groups were introduced into the matching process (age, weight, creatinine; see Table 1).

## 3. Results

### 3.1. Patient Characteristics

A total of 715 out of 1914 patients (37.4%) had a known history of AF and were analysed for the study presented here. A total of 43 patients were treated with the rhythm control strategy (RHY), whereas 672 patients were in the rate control group (RATE) at the time of admission. Table 1 shows the baseline characteristics of these patients.

The data shows that the mean age of patients under rhythm control therapy is significantly lower than in the rate control group. As seen from the table, the mean resting heart rate in RATE is somewhat below the range that is usually considered “adequate” (80/min–110/min). When comparing the two groups, it was found that patients with RATE had a lower mean creatinine value. On the other hand, there are no relevant differences regarding functional status, as seen from MMS and Barthel Index, nor in the majority of the variables examined, respectively. 

### 3.2. Factors Influencing Mini Mental Status

In order to determine which parameters might have an influence on cognitive function as seen from MMS, a linear regression model was applied. First, a number of variables, including age, CHA2DS2-VASc score, HASBLED score, heart rate, creatinine level, and information regarding chronic medical treatment, were examined in a univariate model (Table 2).

The result shows that increasing age as well as a higher serum creatine level and a higher HASBLED (but not CHA_2_DS_2_VAsc) score are linked to an impaired MMS, whereas chronic oral anticoagulation therapy is associated with better scores in the MMS. On the other hand, neither rhythm control therapy nor the other variables studied turned out to be of relevance in this model. In order to control for differences that were found at baseline between RATE and RHY (see Table 1), a propensity score matching was performed were all base line variables that were found to be different between the two groups were introduced into the matching process (age, weight, creatinine). This was leading to 42 matched-pairs of patients, the MMS of these patients is shown in Table 3. The difference in the MMS value in the two subgroups was not statistically significant

Details regarding the antithrombotic therapy of the patients with known history of AF are given in Table 4.

**Figure 1:**
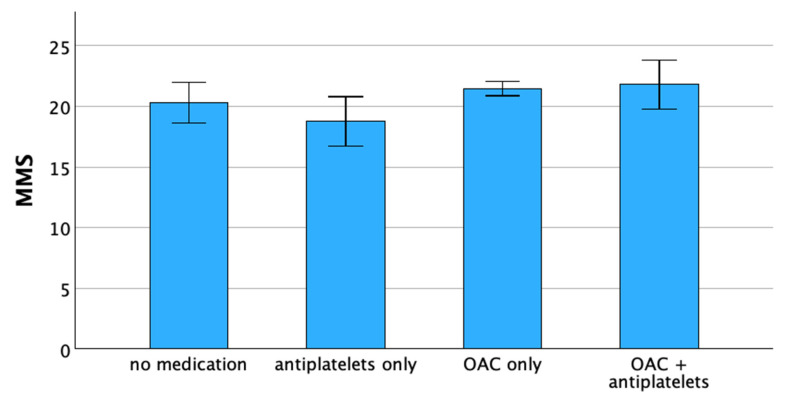
Mini Mental Status in geriatric patients with atrial fibrillation depending on the mode of chronic anticoagulation (*p* < 0.05, ANOVA). Abbreviations: MMS = Mini Mental Status; OAC = oral anticoagulation with NOAC or vitamin K antagonists.

The mean values of the MMS depending on the chosen anticoagulation regime are shown in Figure 1. It can be seen that a higher MMS is found in patients with OAC than in patients without any anticoagulation. However, the lowest values for MMS are found in the group of patients treated with antiplatelet agents only. 

To determine the quantitative effect of the individual anticoagulant regimens on MMS, a logistic regression was used (Table 5). This analysis shows that in geriatric patients with AF, chronic oral anticoagulation is associated with an increased result in the MMS (1.3 points), whereas patients treated with antiplatelets only show a reduction of MMS by 2.6 points.

## 4. Discussion

The typical symptoms of atrial fibrillation, such as palpitations, dizziness and dyspnoea, and even acute heart failure, can significantly reduce the quality of life especially in older patients. Furthermore, regardless of left ventricular ejection fraction, there is an association of AF with mortality and hospital admissions [4]. Concomitant extracardiac diseases such as vascular dementia or depression have also been shown to be associated with AF [5]. Most importantly, AF is the leading cause of ischemic stroke in people over 75 years of age [7], and thromboembolic complications mainly determine the prognosis of patients with AF. In a recent analysis, it has been shown that despite all the therapeutic advances that could be implemented into the therapy of AF within recent years, life expectancy still remains significantly shorter in patients with AF [8]. Therefore, the therapeutic regime is of significant importance, especially in geriatric patients.

There are different therapeutic pathways that can be followed in the treatment of patients with AF. Often a combination of drug therapy and interventional procedures such as pulmonary vein isolation is useful. The effective prevention of embolic complications has to be regarded as the essential part in the treatment of AF patients. Systemic anticoagulation nowadays is still the standard of care with a proven life-prolonging effect for patients with AF [10], although interventional occlusion of the left atrial appendage has become a valuable alternative in this regard, especially for patients with a high bleeding risk [20,21,22].

The treatment of atrial fibrillation should be based on multimodal decisions taking into account the existing symptoms, the duration of the disease, risk factors regarding both thromboembolic and bleeding complications, respectively, as well as the age and concomitant diseases of the individual patients. In correspondence to other diseases, it can also be assumed for atrial fibrillation that, especially in geriatric patients, a notable discrepancy between guideline recommendations and treatment practice can be observed, as it has already been shown, for example, for osteoporosis [23] or for heart failure [24].

Our prospective observational study describes the situation of geriatric patients with AF. Regardless of the treatment strategy (rhythm vs. rate control), a comparable proportion of patients in both groups received oral anticoagulation (81.4% vs. 82.3%, respectively). Patients under rhythm control were found to be younger than in the rate control group, which is in good agreement with previously collected data [25]. In our study, there were no relevant differences in other clinical variables between the two treatment groups. The tendency to have a higher rate of previous strokes in the rate control group might be due to the higher patient age.

It is well known that the frequency of AF increases with age in men and women [26]. The prevalence of 37.4%, as seen in the data presented here [19], fits well into the international surveys of geriatric patients conducted so far [27,28]. 

In our study, 84% of patients with known AF are effectively anticoagulated. This proportion is significantly higher than in previous publications (17%, 64.2%, 63% [27,28,29]), although a considerable proportion of 16% of patients is still not adequately protected against thromboembolic complications. Contraindications for OAC were not explicitly evaluated in the study protocol, especially as, according to our current understanding, this assessment in the majority of cases is more based on a patient-specific individual risk-benefit evaluation by the treating physicians than on a defined catalogue of diagnoses. However, as described before, our analysis shows that 28.1% of the patients with AF and without an existing OAC had a bleeding anamnesis [19]. The HAS-BLED score in contrast was higher in the anticoagulated patients than in the non-anticoagulated patients and thus cannot be used as an explanation for not taking OACs in the analysed patient population. As a bottom line, it can be assumed that the availability of NOAKs in particular has led to a more consistent implementation of the anticoagulation recommendations compared to earlier studies [30].

One focus of the present study was to identify factors that influence the Mini Mental Status (MMS) as a measure of cognitive impairment in geriatric patients. Our regression analyses showed that increasing age and decreasing kidney function are associated with lower results in MMS. Surprisingly, the patients’ risk of bleeding (quantified by the HASBLED score) rather than the CHA_2_DS_2_-VASc score, was also associated with a decline in MMS. The only variable for which a positive association with MMS could be demonstrated in these patients with AF was the presence of chronic oral anticoagulation therapy. These data suggest that inhibition of thrombus formation in geriatric AF patients exerts a favourable effect on the MMS and thus might have a protective effect regarding the development of dementia, as postulated before [6]. In concordance, Ding et al. also came to the conclusion that the risk of dementia can be reduced by effective anticoagulation after a 9-year observation period [25].

Perhaps even more important, our data clearly indicates that treatment with antiplatelets seems to have an unfavourable effect on cognitive function in geriatric patients with AF: the presence of chronic antiplatelet medication was associated with the lowest MMS values (reduction by 2.6 points). In other words, even those patients who received no antithrombotic medication at all showed better MMS values than the patients under antiplatelets. This again emphasizes that antiplatelets should not be considered an “alternative treatment” in patients with AF and contraindications regarding OAC [5].

## 5. Conclusions

In our analysis, it was not possible to demonstrate any influence of rhythm control (RHY) or rate control (RATE) strategies on the cerebral performance of geriatric patients with AF. However, the use of oral anticoagulation is clearly associated with better results in the Mini Mental Status, whereas antiplatelets cannot be considered a reasonable therapeutic alternative in these patients.

### Strengths and Weaknesses of the Study

This is a prospective study, and due to the comparatively large number of cases, the data can be considered robust. However, it must be noted that, due to the observational design of the study, only statistical associations can be described without providing evidence of a causal relationship. Detailed information regarding the duration of AF could not be collected and therefore not included in the analysis. Additionally, due to the nature of the study, no information regarding mortality, thromboembolic events, or bleeding rates can be given, respectively, as all information in the study was taken from a single time point. The rather low number of patients in RHY has to be considered a limitation as well.

## Figures and Tables

**Table 1:** Baseline characteristics of geriatric patients with AF at study inclusion.

Variable	Patients with Rhythm Control	Patients with Frequency Control	*p*-Value
number	43 (6%)	672 (94%)	-
age (years)	79.7 ± 5.8	84 ± 5.8	<0.001
female gender	28 (65.1%)	444 (66.1%)	0.898
height (cm)	167 ± 9	166 ± 9	0.581
weight (kg)	84.8 ± 16.3	76.8 ± 25.9	0.046
Barthel index	36 ± 12	34 ± 13	0.374
MMS	22 ± 7	21 ± 7	0.398
CHA2DS2Vasc	3.86 ± 1.06	4.18 ± 1.16	0.082
HASBLED	3.42 ± 0.91	3.56 ± 0.90	0.334
heart rate (bpm)	73 ± 19	75 ± 15	0.527
creatinine (µmol/L)	126 ± 48	105 ± 51	0.008
ALAT (µmol/L)	0.44 ± 0.28	0.45 ± 0.70	0.904
ASAT (µmol/L)	0.54 ± 0.36	0.54 ± 0.57	0.994
bilirubin (µmol/L)	13 ± 13	12 ± 7	0.704
hypertension	34 (79.1%)	579 (86.2%)	0.197
diabetes	5 (11.6%)	79 (11.8%)	0.98
heart failure	32 (74.4%)	421 (62.6%)	0.12
previous stroke	9 (20.9%)	194 (28.9%)	0.263
any antiplatelet medication	7 (16.3%)	84 (12.5%)	0.49
aspirin	4 (9.3%)	62 (9.2%)	0.987
clopidogrel	5 (11.6%)	34 (5.1%)	0.066
any OAC	35 (81.4%)	553 (82.3%)	0.881
VKA	2 (4.7%)	52 (7.7%)	0.458
dabigatran	0 (0%)	28 (4.2%)	0.172
rivaroxaban	5 (11.6%)	98 (14.6%)	0.593
apixaban	18 (41.9%)	245 (36.5%)	0.476
edoxaban	10 (23.3%)	133 (19.8%)	0.582
CCB	10 (23.3%)	204 (30.4%)	0.324
beta blockers	31 (72.1%)	515 (76.6%)	0.497
ACE inhibitor	28 (65.1%)	402 (59.8%)	0.492
digitalis glycosides	4 (9.3%)	98 (14.6%)	0.337
amiodarone	33 (76.7%)	0 (0%)	<0.001
history of ablation	10 (23.3%)	0 (0%)	<0.001
LAAC	2 (4.7%)	12 (1.8%)	0.189

Abbreviations: MMS = Mini-Mental Status; bpm = beats per minute; ALAT = alanine aminotransferase; ASAT = aspartate aminotransferase; OAC = oral anticoagulation; VKA: vitamin K antagonist oral anticoagulation; CCB = calcium channel blocker; ACE = angiotensin-converting enzyme; LAAC = left atrial appendage closure.

**Table 2:** Influence of different variables on Mini Mental Status (univariate linear regression analysis).

Variable	Regression Coefficient B	*p*-Value
age (years)	−0.212	<0.001
CHA_2_DS_2_Vasc	−0.396	0.088
HASBLED	−0.881	0.003
OAC	1.832	0.008
creatinine (µmol/L)	−0.011	0.03
CCB	0.067	0.908
beta blockers	0.434	0.488
digitalis glykosides	−0.573	0.462
amiodarone	1.594	0.193
heart rate (bpm)	0.007	0.768
rhythm control	0.91	0.398

Abbreviations: OAC = oral anticoagulation with NOAC or vitamin K antagonists; CCB = Calcium channel blockers.

**Table 3:** Comparison of Mini Mental Status (MMS) in the two groups RHY and RATE after propensity score matching using the baseline variables age, weight, and creatinine.

Variable	RHY (*n* = 42)	RATE (*n* = 42)	*p*-Value
MMS	22.0 ± 7.4	19.9 ± 7.3	0.20

**Table 4:** Antithrombotic medication in geriatric patients with atrial fibrillation.

Total	No Anticoagulation	Antiplatelet Medication Only	OAC Only	Combination of Antiplatelets and OAC
715 (100%)	76 (10.6%)	51 (7.1%)	541 (75.7%)	47 (6.6%)

Abbreviations: OAC: oral anticoagulation with NOAC or VKA.

**Table 5:** Influence of the type of anticoagulation on the Mini Mental Status in geriatric patients with atrial fibrillation.

Variable	Regression Coefficient B	*p*-Value
no anticoagulation	−0.974	0.258
antiplatelets only	−2.621	0.01
OAC only	1.262	0.042
OAC + antiplatelets	0.669	0.544

Abbreviations: OAC = oral anticoagulation with NOAC or vitamin K antagonists.

## Data Availability

All data can be obtained from the authors on reasonable request.
